# p90 ribosomal S6 kinase (RSK) phosphorylates myosin phosphatase and thereby controls edge dynamics during cell migration

**DOI:** 10.1074/jbc.RA119.007431

**Published:** 2019-05-28

**Authors:** Shiela C. Samson, Andrew Elliott, Brian D. Mueller, Yung Kim, Keith R. Carney, Jared P. Bergman, John Blenis, Michelle C. Mendoza

**Affiliations:** From the ‡ Department of Oncological Sciences, Huntsman Cancer Institute, University of Utah, Salt Lake City, Utah 84112 and; the § Department of Cell Biology, Harvard Medical School, Boston, Massachusetts 02115

**Keywords:** extracellular-signal-regulated kinase (ERK), myosin, cell migration, cell signaling, cytoskeleton, cell motility, myosin phosphatase targeting subunit (MYPT1), p90 ribosomal S6 kinase (RSK), Rho kinase (ROCK)

## Abstract

Cell migration is essential to embryonic development, wound healing, and cancer cell dissemination. Cells move via leading-edge protrusion, substrate adhesion, and retraction of the cell's rear. The molecular mechanisms by which extracellular cues signal to the actomyosin cytoskeleton to control these motility mechanics are poorly understood. The growth factor-responsive and oncogenically activated protein extracellular signal-regulated kinase (ERK) promotes motility by signaling in actin polymerization-mediated edge protrusion. Using a combination of immunoblotting, co-immunoprecipitation, and myosin-binding experiments and cell migration assays, we show here that ERK also signals to the contractile machinery through its substrate, p90 ribosomal S6 kinase (RSK). We probed the signaling and migration dynamics of multiple mammalian cell lines and found that RSK phosphorylates myosin phosphatase–targeting subunit 1 (MYPT1) at Ser-507, which promotes an interaction of Rho kinase (ROCK) with MYPT1 and inhibits myosin targeting. We find that by inhibiting the myosin phosphatase, ERK and RSK promote myosin II–mediated tension for lamella expansion and optimal edge dynamics for cell migration. These findings suggest that ERK activity can coordinately amplify both protrusive and contractile forces for optimal cell motility.

## Introduction

Cells move via cycles of edge protrusion, focal adhesion formation, and cell body translocation (1, 2). Sustained edge protrusion, along with tail retraction, drives migration speed and directionality for proficient movement. In epithelial cells, edge protrusion is powered by a dendritically branched network of actin filaments growing against the plasma membrane (3, 4). 1–4 μm back from the cell edge, in a structure called the lamella, myosin II motors pull on the actin network to generate the traction force and tension necessary for persistent protrusions and periodic edge retraction (4–8). The contractile actomyosin structures couple to focal adhesions and are generated from bundles of actin filaments (9–14). Signaling pathways impinge on these mechanical processes to regulate their timing and power.

The ERK^3^ signaling pathway controls cell proliferation, survival, and motility in response to extracellular cues (15–17). ERK is activated by growth factor signals to the small GTPase RAS, which then activates the kinase RAF, which phosphorylates and activates the kinase MEK, which activates ERK (18, 19). ERK directly regulates the actin polymerization machinery to stabilize protrusions into fast, prolonged events with the power to overcome membrane tension and promote motility (20, 21). It stands to reason that increased protrusion power must be accompanied by modulation of adhesion and contraction dynamics for productive motility. Indeed, ERK has been found to promote adhesion disassembly (22, 23) and myosin activity (24–26), but the mechanisms and molecular roles of this regulation in cell migration are unknown.

Myosin II motors contain heavy chains with ATPase domains and light chains (MLCs) that must be phosphorylated for activity (14). Myosin light chain kinase (MYLK) and myotonic dystrophy kinase-related Cdc42-binding kinase phosphorylate MLC (14). Myosin light chain phosphatase (MLCP) dephosphorylates and inactivates MLC. MLCP is comprised of the PP1Cβ catalytic subunit and the myosin phosphatase-targeting subunit (MYPT1) (14, 27). Rho kinase (ROCK) induces myosin activity by phosphorylating MYPT1 at Thr-696 and Thr-853 (14, 28, 29). Thr-696 phosphorylation inhibits MLCP catalytic activity toward myosin II, whereas Thr-853 phosphorylation inhibits MLCP interaction with myosin II (30–32).

MLC phosphorylation in the leading edge of migrating cells is dynamic. Phosphorylation and myosin activation occurs at the tip of protruding edges and increases during an initial slow phase of retraction (33). MLC is then dephosphorylated as edge retraction rate and magnitude increases (33). MYLK and ROCK have partially overlapping functions in these edge dynamics and cell migration. MYLK is activated in and required for protrusions and the initial slow phase of retraction (33–36). ROCK regulates myosin further back from the cell edge, promoting adhesion stabilization as the cell moves forward and also cell body translocation (34–36). MYLK and MYPT1 inhibition block migration persistence, a measurement of how well cells maintain their direction of motion. ROCK inhibition increases persistence (35, 37). An optimal balance of MYLK-phosphorylated and MLCP-dephosphoryated MLC pools enables migration persistence.

ERK can directly phosphorylate and activate MYLK *in vitro*, suggesting a mechanism by which ERK might regulate myosin contractility (15, 25). However, optimal and conserved ERK phospho-motifs are not found in MYLK (38), and no site has been identified or validated in ERK substrate screens (39–41). Here, we sought to uncover the signaling mechanism by which ERK modulates myosin during cell migration. We found that the ERK-regulated p90 ribosomal S6 kinase (RSK) phosphorylates MYPT1 at Ser-507, and this promotes inhibitory phosphorylations on MLCP, cell motility, and lamella edge dynamics.

## Results

To identify ERK-mediated myosin II regulatory mechanisms, we searched for phospho-signals to the myosin machinery that might be mediated by RSK. RSK is a member of the AGC kinase family. This family also includes AKT and S6K within the mTORC1 signaling pathway and mitogen- and stress-activated protein kinases (MSKs) downstream of ERK and p38/c-Jun N-terminal kinase MAP kinases (42, 43). These kinases exhibit promiscuity in targeting R*X*R*XX*(pS/pT) motifs (43, 44), although the RSKs do not strictly require the −5 Arg (45). Screens for AGC kinase substrates have identified phosphorylation sites on MYPT1 at Ser-507 and Ser-668 (40). The Ser-507 site (PRRLApS) follows the less-stringent R*XX*pS phospho-motif and has been identified more than 800 times in high throughput phosphorylation studies, more than any other MYPT1 phosphosite (www.phosphosite.org; Ref. 92).^4^ For example, Ser(P)-507 was induced upon insulin stimulation of CHO cells overexpressing insulin receptor and L6K76 myoblasts (46, 47). The Ser-668 site (RERRRpS) follows the more stringent AGC motif. Ser-668 has been characterized as a PKG substrate and also suggested, but not formally tested, to function as a RSK substrate (26, 48). Both sites are conserved in vertebrates (www.phosphosite.org).

**Figure 1.**
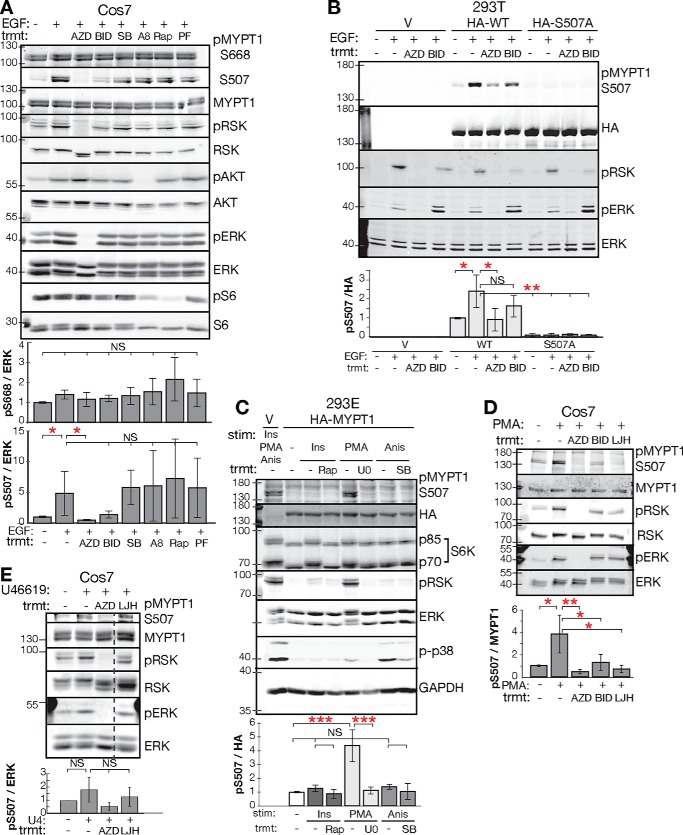
**RSK phosphorylates MYPT1 Ser-507.** *A*, Western blot and quantification of endogenous MYPT1 phosphorylation in Cos7 cells. pMYPT1 S507/total ERK and pMYPT1 Ser-668/total ERK are relative to the signal in the starved condition without stimulation (*Stim*, normalized to 1.0). ERK's distinct protein size allows for simultaneous probing of p-MYPT1 on the same membrane. *n* = 4 biological replicates for pMYPT1 Ser-668, and *n* = 3 biological replicates for pMYPT1 S507. *B* and *C*, Western blot and quantification of exogenous MYPT1 S507 phosphorylation in 293T and 293E cells. *V* is empty vector control transfection. HA-WT and HA-S507A are HA-tagged WT and S507A mutant transfections, respectively. pMYPT1 S507/HA is relative to the signal in the HA-WT starved condition. *n* = 3 biological replicates each. Endogenous phospho-MYPT1 is not detected in the vector transfection conditions because of the reduced intensity used to scan the overexpressed HA-MYPT1 Western blots. *D* and *E*, Western blots of endogenous MYPT1 S507 phosphorylation in Cos7 cells. pMYPT1 S507 is relative to the signal in the starved condition. *n* = 3 biological replicates. One lane of irrelevant treatment condition uniformly removed from *E*, marked by a *dashed line. Error bars* for Western blotting quantifications indicate S.D. The pathway agonists are: EGF, insulin (*Ins*), PMA, anisomycin (*Anis*), and U46619 thromboxane agonist (*U4*). The inhibitors are MEK inhibitors AZD6244 (*AZD*) and U0126 (*U0*), RSK inhibitors BI-D1870 (*BID*), and LJH685 (*LJH*), p38 inhibitor SB203580 (*SB*), AKT inhibitor AKTVIII (*A8*), mTORC1 inhibitor rapamycin (*Rap*), and S6 kinase inhibitor PF-4708671 (*PF*). *p-RSK* is p-RSKT359,S363. *p-AKT* is p-AKT Ser-473. One-way ANOVA was used. *, *p* < 0.05; **, *p* < 0.01; ***, *p* < 0.001; *NS*, not significant (*p* > 0.05); *trmt*, treatment.

We tested whether RSK phosphorylates MYPT1 Ser-507 or Ser-668, using commercially available antibodies. We used 293T cells, which respond to epidermal growth factor (EGF) stimulation by activating the RAS/ERK and AKT/mTORC1/S6K pathways. MYPT1 Ser-507 phosphorylation trended to increase with EGF stimulation and decrease upon MEK inhibition with AZD6244 and RSK inhibition with BI-D1870. Ser-668 did not exhibit phospho-regulation (Fig. S1*A*). The mTORC1 and S6K1 inhibitors rapamycin and PF-4708671 did not affect the phosphorylation of either site. In Cos7 cells, similar to 293T cells, EGF induced MYPT1 Ser-507, but not Ser-668, phosphorylation (Fig. 1*A*). The Ser-507 phosphorylation was sensitive to both MEK (one-way ANOVA, *p* = 0.03) and RSK inhibition (trend, *p* = 0.10), but not AKT or S6K inhibition (Fig. 1*A*). We additionally tested p38/c-Jun N-terminal kinase inhibition with SB203580 to assess contribution from MSKs and found no effect on the phosphorylation of either site (Fig. 1*A*). Thus, in cells capable of signaling through multiple growth factor pathways, MYPT1 Ser-507 appears to be targeted by RSK and regulated by the RAS/RAF/MEK/ERK pathway.

Because the phospho-MYPT1 Ser-507 antibody nonspecifically detects a protein larger than MYPT1 in endogenous lysate, we validated the antibody using phosphosite mutants. We transfected 293T cells with HA-tagged MYPT1 WT and mutant MYPT1 S507A (Fig. 1*B*). The phospho-MYPT1 Ser-507 antibody recognized a single EGF-stimulated band (one-way ANOVA, *p* = 0.03) that was reduced with MEK inhibition (*p* = 0.02), trended lower with RSK inhibition (*p* = 0.5), and completely abrogated with the S507A mutant (*p* < 0.002), confirming its specificity.

Previous studies in insulin-sensitive cell models suggest that AKT and/or S6K may contribute to MYPT1 Ser-507 in some cases (46, 47). Our results suggest that MEK may additionally signal to MYPT1 Ser-507 independent of RSK, because MEK inhibitors more completely blocked MYPT1 Ser-507 phosphorylation than RSK inhibitors (Fig. 1, *A* and *B*, and Fig. S1*A*). It is possible that with EGF stimulation, ERK-mediated activation of mTORC1 signaling leads to S6K activation and additional phosphorylation of MYPT1 (44). We therefore further tested RSK's role as the major MYPT1 Ser-507 kinase with distinct pathway agonists and inhibitors in multiple cell types. Using 293E cells, we induced ERK/RSK activity with phorbol 12-myristate 13-acetate (PMA). PMA directly activates PKC, which phosphorylates and activates RAF for ERK/RSK activation (49–53). PMA induced Ser-507 phosphorylation (one-way ANOVA, *p* = 0.00003), and the MEK inhibitor U0126 completely blocked the induction (*p* = 0.00004; Fig. 1*C*). Neither insulin or anisomycin, which activate Akt/mTOR/S6K and p38/MSK, respectively, regulated Ser-507 phosphorylation (Fig. 1*C*). PMA also induced MYPT1 Ser-507 phosphorylation in Cos7 cells (one-way ANOVA, *p* = 0.01), and in this case, the phosphorylation was sensitive to the RSK inhibitor BI-D1870 (*p* = 0.03) and a structurally distinct RSK inhibitor LHJ685 (*p* = 0.03; Fig. 1*D*). Further, treatment of Cos7 cells with the thromboxane mimetic U46619 also induced MYPT1 Ser-507 phosphorylation (trend, *p* = 0.6; Fig. 1*E*). U46619 acts through the thromboxane G protein–coupled receptor to activate the ERK- and p38-MAPKs and ROCK (26, 54, 55). MYPT1 Ser-507 phosphorylation trended lower with MEK and RSK inhibition, again suggesting that it is mediated by RSK (Fig. 1*E*).

We tested regulation of MYPT1 Ser-507 in four additional cell lines: untransformed kidney (MDCK) cells, human and murine non-small cell lung cancer lines (A549 and 3658) (56), and human fibrosarcoma (HT1080). Again, PMA-induced MYPT1 Ser-507 phosphorylation, and this was sensitive to both MEK and RSK inhibition (Fig. S1*B*). These data with multiple ERK/RSK pathway agonists and inhibitors, in multiple cell lines, indicate that RSK phosphorylates MYPT1 Ser-507 and that in systems with multiple operative growth factor signaling pathways, RSK is the dominant AGC kinase for this site.

No agonist or inhibitor reproducibly altered Ser(P)-668 levels detected by Western blotting of radioimmune precipitation assay buffer or 10% TCA lysates (Fig. 1*A*, Fig. S1*A*, and data not shown). However, the phospho-antibody has been used to detect a specific signal in HeLa and Jurkat cells lysed with the calyculin A phosphatase inhibitor and in smooth muscle cells stimulated with U46619 (26, 57). We therefore tested the anti–phospho-MYPT1 Ser-668 antibody with staurosporine, a pan-kinase inhibitor. Staurosporine blocked phospho-ERK but had no effect on the signal from the phospho-MYPT1 Ser-668 antibody (Fig. S1*C*). We conclude that the MYPT1 Ser-668 phosphosite is poorly regulated by growth factor-activated kinases, and regulation is rather likely driven by phosphatases.

**Figure 2.**
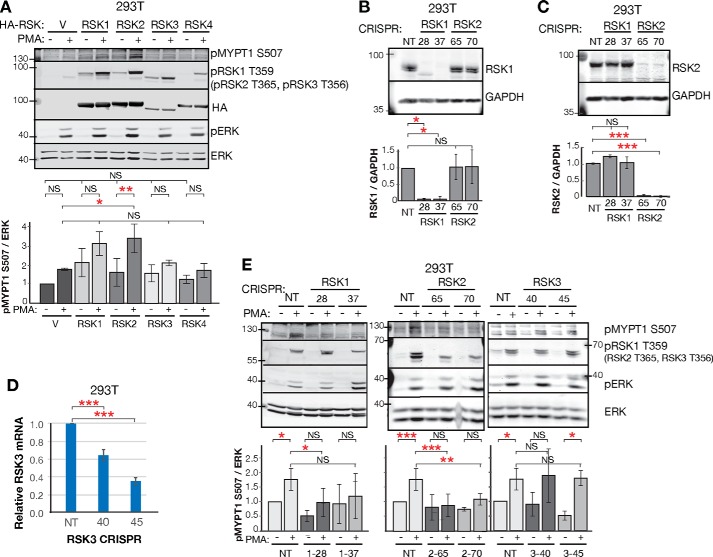
**RSK1 and RSK2 phosphorylate MYPT1 Ser-507.** *A*, Western blot and quantification of MYPT1 Ser-507 phosphorylation in 293T cells upon RSK homolog overexpression. pMYPT1 Ser-507/ERK signal is relative to the vector + starved condition (normalized to 1.0). *n* = 5 biological replicates. *Error bars* indicate S.D. Endogenous phospho-RSK is not detected in the vector control because of the reduced intensity used to scan Western blots with overexpressed HA-RSK. *B* and *C*, Western blot and quantification of RSK1 and RSK2 expression in CRISPR knockout clones, generated with gRNAs RSK1-28 and RSK1-37 to knockout RSK1 and gRNAs RSK2-65 and RSK2-70 to knockout RSK2. *n* = 3 biological replicates. *Error bars* indicate S.D. RSK/GAPDH signal is relative to that in the nontargeting control CRISPR (*NT*) gRNA control condition (normalized to 1.0). *D*, RT-PCR of RSK3 expression in CRISPR knockout clones. RSK3 mRNA levels are normalized to GAPDH mRNA. *n* = 3 biological replicates. *Error bars* indicate S.E. with four technical replicates per experiment. *E*, Western blot and quantification of MYPT1 Ser-507 phosphorylation in 293T cells upon RSK knockout. pMYPT1S507/ERK signal is relative to the NT + starved condition (normalized to 1.0). *n* = 3 biological replicates. *Error bars* indicate S.D. *V*, empty vector control transfection. One-way ANOVA was used. *, *p* < 0.05; **, *p* < 0.01; ***, *p* < 0.001; *NS*, not significant (*p* > 0.05).

Because the RSK homologs may have distinct effects on cell motility (58–62), we sought to determine which RSK homologs phosphorylate MYPT1. RSK1–3 are expressed ubiquitously and their activity is induced by growth factor activation of the RAS/ERK pathway (42, 63). RSK4 expression is limited to embryonic development (63). We overexpressed RSK1–4 into 293T cells and assayed endogenous MYPT1 phosphorylation. RSK activation was confirmed with an antibody against pRSK T359 (RSK1) that recognizes the phosphorylated epitope in all RSK homologs. RSK1 expression trended to increase, and RSK2 expression increased PMA-induced stimulation of MYPT1 Ser-507 (one-way ANOVA vector + PMA *versus* RSK1 + PMA, *p* = 0.07; and RSK2 + PMA, *p* = 0.02; RSK2 + no stimulation *versus* RSK2 + PMA, *p* = 0.008; Fig. 2*A*). Thus, both RSK1 and RSK2 likely phosphorylate Ser-507, although RSK2 may be the more dominant kinase. Despite transfection optimization, we were unable to express RSK3 and 4 to the same level as RSK1 and RSK2. We then tested the contribution of RSK1, RSK2, and RSK3 to MYPT1 Ser-507 phosphorylation by CRISPR knockout. Two distinct CRISPR gRNAs were designed for RSK1–3 (RSK1-28 and RSK1-37, RSK2-65 and RSK2-70, and RSK3-40 and RSK3-45). We generated single-cell clones with each sgRNA and confirmed loss of RSK1 and RSK2 expression by Western blotting (Fig. 2, *B* and *C*) and RSK3 by real-time PCR (Fig. 2*D*). Mutation within the target site was confirmed by sequencing (Fig. S1*D*). The RSK1 and RSK2 knockouts exhibited reduced PMA-stimulated Ser-507 phosphorylation (one-way ANOVA with RSK1-28, *p* = 0.04; trend for RSK1-37, *p* = 0.3; RSK2-65, *p* = 0.0002; and RSK2-70, *p* = 0.01; Fig. 2*E*). However, the phosphosignal was not abrogated in either case, suggesting that in the absence of RSK1, RSK2 continues to phosphorylate MYPT1 Ser-507, and in the absence of RSK2, RSK1 continues to phosphorylate MYPT1 Ser-507. RSK3 knockout had little effect on MYPT1 Ser-507 phosphorylation. Together, the overexpression and knockout experiments indicate that RSK1 and RSK2 are the major MYPT1 Ser-507 kinases.

**Figure 3.**
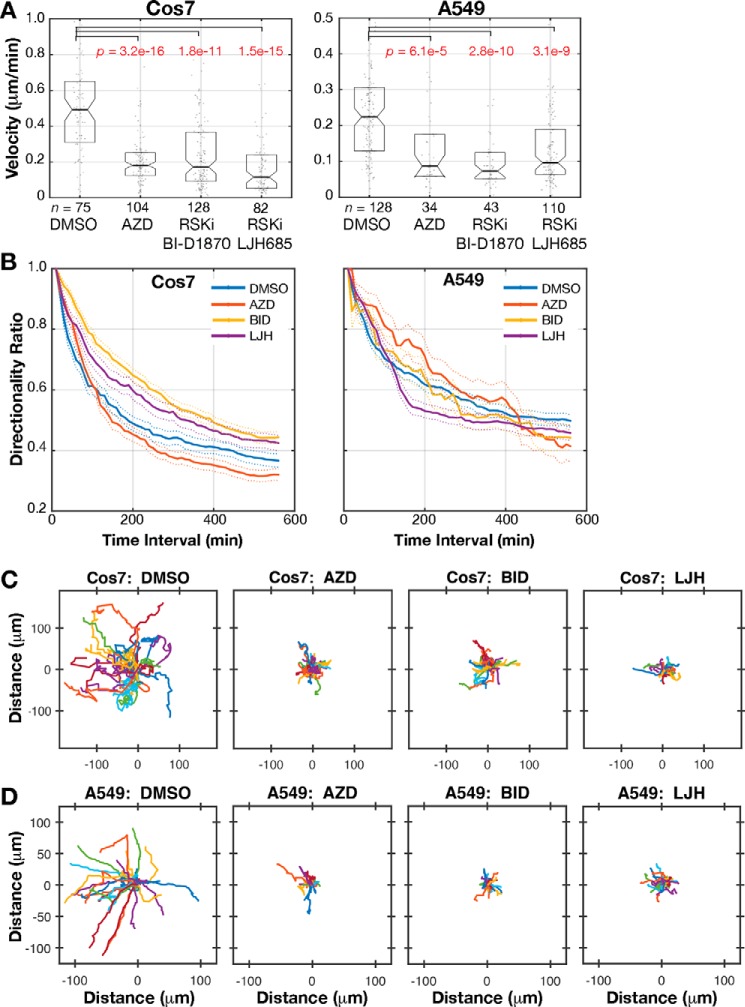
**RSK controls cell motility.** *A*, cell migration velocity distributions of *n* Cos7 and A549 cells treated with DMSO, MEK inhibitor AZD6244 (*AZD*), or RSK inhibitor BI-D1870 (*BID*) or LJH685 (*LJH*). The cells were tracked from at least three independent experiments. Each data point is the mean velocity for a cell. *Boxes* span the 25^th^ to 75^th^ distribution. The *central horizontal line* indicates the median for all cells. *Notches* indicate 95% CI around the median. *p* values in *red* show samples with distributions distinct from the control DMSO condition, from Kolmogorov–Smirnov test. *B*, mean directionality of cells analyzed in *A*, plotted for each time interval. *Dashed lines* show S.E. *C*, plots of migrating Cos7. *D*, A549 cells. Tracks of the 25 cells with migration speed closest to the median are depicted.

RSK activity is reported to be necessary and sufficient for cell migration, based on studies with RSK inhibitors SL0101, FMK, and BI-D1870 and constitutively active RSK1 and RSK2 in HeLa, MCF10a mammary epithelial, and WM35 melanoma cells (58, 61, 62). However, a conflicting report with RSK1 siRNA suggests that RSK1 inhibits migration in nonsmall cell lung cancer cells, including A549 cells (60). We sought to determine whether overall RSK activity promotes or inhibits migration using a random-walk assay with the migratory Cos7 and A549 cell lines. We manually tracked the migration paths over 4–6 h and calculated velocity (average displacement for a 10-min time interval) and persistence (ratio of displacement to trajectory length). As expected, MEK inhibition with AZD6244 reduced migration velocity and path length (Fig. 3, *A*, *C*, and *D*). Inhibition with RSK inhibitors BI-D1870 and LJH685 also blocked migration. Cos7 cell median migration speed of 0.492 μm/min with DMSO treatment was reduced to 0.172 and 0.116 μm/min with RSK inhibitors BI-D1870 and LJH685 (*p* = 1.8E-11 and *p* = 1.5E-15, two-sample nonparametric Kolmogorov–Smirnov test; Fig. 3*A*). A549 cell median migration speed of 0.224 μm/min with DMSO treatment was reduced to 0.074 and 0.096 μm/min with RSK inhibitors BI-D1870 and LJH685, *p* = 2.8E-10 and *p* = 3.1E-9; Fig. 3*A*. We tested the migration of three additional cell lines (MDCK, 3658, and HT1080), and all exhibited reduced migration velocity upon MEK and RSK inhibition (Fig. S2).

A directional persistence ratio of 1.0 indicates movement in a straight line, whereas a ratio closer to 0 indicates motility with frequent turns. In Cos7 cells, MEK inhibition reduced persistence, whereas RSK inhibition with either BI-D1870 or LJH685 increased persistence (Fig. 3*B*). MEK/RSK inhibition did not affect persistence in A549 cells. Similarly, in MDCK and 3658 cells, MEK inhibition reduced persistence, whereas RSK inhibition increased persistence (Fig. S2, *B* and *E*). Pathway inhibition did not affect persistence in HT1080 cells (Fig. S2*H*, directionality profiles exhibit overlapping S.E.). These data suggest that RSK activity promotes migration velocity and, in some cells types, inhibits directionality. The finding that ERK promotes but RSK inhibits persistence is likely due to ERK's regulation of additional motility effectors beyond RSK, such as the WAVE regulatory complex (20).

**Figure 4.**
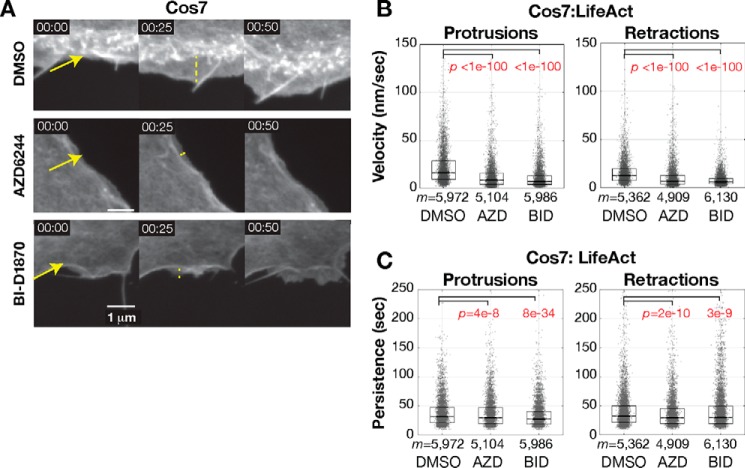
**RSK controls cell edge dynamics.** *A*, representative images of migrating Cos7 cells at 25-s intervals. *Arrows* show region of interest that protrudes in later frames. The *dashed line* shows protrusive region. *B*, protrusion and retraction velocity distributions. *C*, protrusion and retraction persistence distributions of *m* significant protrusion events in *n* = 6 cells treated with DMSO, 5 cells with AZD6244 (*AZD*), and 6 cells with BI-D1870 (*BID*). *Boxes* span the 25^th^ to 75^th^ distribution. The *central horizontal line* indicates the median. Notches are 95% CI of median. Samples with distributions distinct from control have *p* values labeled in *red*, *p* value from Kolmogorov–Smirnov test. Inhibitors are the MEK inhibitor AZD6244 and the RSK inhibitor BI-D1870.

We tested whether RSK is required for myosin II–mediated edge dynamics during cell migration. We assayed Cos7 cells, which exhibit canonical protrusion–retraction cycles during migration (7). We transfected cells with the actin cytoskeleton marker Emerald-LifeAct and imaged their steady-state dynamics. In DMSO-treated cells, an area of low actin density expanded behind the edge with treadmilling actin during protrusion (Fig. 4*A*, *DMSO*). In inhibitor-treated cells, the ruffling edge abutted the stable actin meshwork of the cell body, suggesting minimal lamella expansion (Fig. 4*A*, *AZD* and *BI-D*). We identified and tracked the cell edge over time using MATLAB (21, 64). We detected protrusion and retraction events and calculated their velocities. We found that MEK and RSK inhibition dramatically reduced protrusion and retraction velocity (∼50% reduction) and had a small effect on protrusion persistence (*p* < 0.05, Kolmogorov–Smirnov test; Fig. 4, *B* and *C*). Median protrusion velocity of 16.4 nm/s in DMSO-treated cells was reduced to 8.8 and 7.2 nm/s with MEK and RSK inhibition (Fig. 4*B*). Median retraction velocity of 12.8 nm/s in DMSO-treated cells was reduced to 7.0 and 6.2 nm/s with MEK and RSK inhibition (Fig. 4*B*). A similar 50% reduction was found in the 25% fastest events (Fig. S3*A*).

We repeated this experiment with an independent end user and using the structurally distinct actin probe F-tractin, which exhibits less background binding to unpolymerized actin and distinct effects on native actin dynamics (65). On two distinct days, control cells treated with DMSO exhibited very similar velocity dynamics (DMSO1 and DMSO2; Fig. S3*B*). Again, MEK and RSK inhibition with AZD6244 and the second RSK inhibitor LJH685 reduced edge protrusion and retraction velocities (Fig. S3*B*).

MEK and RSK inhibition nominally reduced protrusion and retraction persistence. Mean protrusion duration of 31.2 s in DMSO-treated cells was reduced to 29.4 and 27.5 s with MEK and RSK inhibition (*p* < 0.05, Kolmogorov–Smirnov test; Fig. 4*C*). Retraction persistence of 32.3 s in DMSO-treated cells was reduced to 29.3 and 30.0 s with MEK and RSK inhibition (*p* < 0.05, Kolmogorov–Smirnov test; Fig. 4*C*). Taken together, these protrusion and retraction analyses indicate RSK signaling promotes lamella expansion and edge velocity and persistence for productive migration.

**Figure 5.**
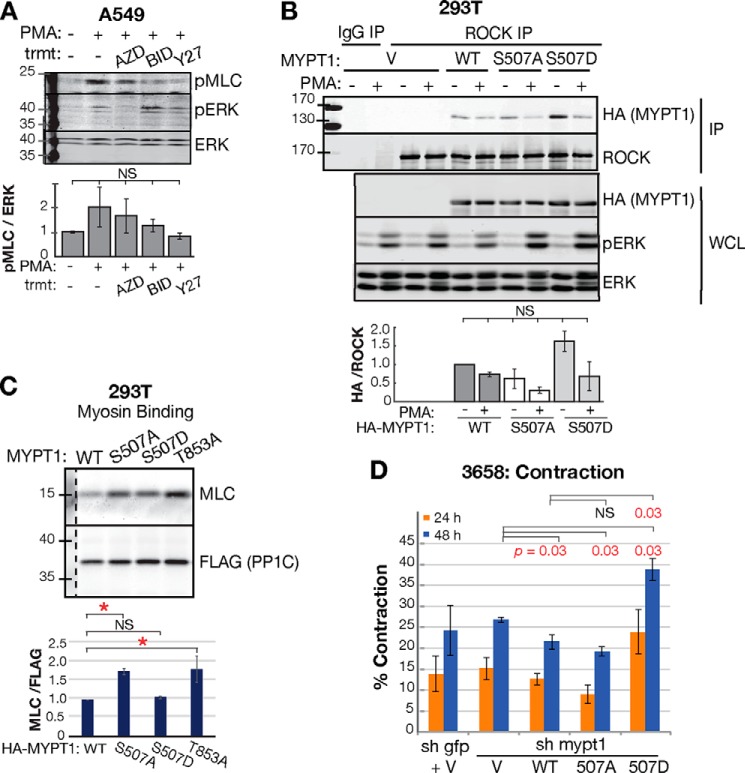
**RSK phosphorylation of MYPT1 promotes ROCK interaction with MYPT1 and inhibition of MLCP.** *A*, Western blot and quantification of MLC phosphorylation in A549 cells. pMLC/ERK signal is relative to the starved condition (normalized to 1.0). *n* = 3 biological replicates. *B*, co-IP and quantification of HA-MYPT1 with ROCK in 293T cells. Whole cell lysate (*WCL*) samples were loaded next to the IP samples in the same gel and show equal input loading of IP and pathway signaling. One molecular weight ladder next to the IP was used to identify the molecular weight. HA-MYPT1/ROCK is relative to the HA-MYPT1 WT starved condition (normalized to 1.0). *n* = 3 biological replicates. *C*, *in vitro* myosin binding of IP'd MPYT1/PP1C complexes and full-length myosin. The *dashed line* marks where one lane of irrelevant treatment condition was uniformly removed from the blots. *n* = 2 biological replicates. One-way ANOVA was used. *, *p* < 0.05; **, *p* < 0.01; ***, *p* < 0.001; *NS*, not significant (*p* > 0.05). *D*, collagen contraction by 3658 cells with stable MYPT1 manipulation. *Error bars* show S.E. for four independent experiments. No manipulations were significant within the first 24 h. Medians distinct from control in 48 h are denoted with *p* value in *red* (Mann–Whitney test). The inhibitors are MEK inhibitor AZD6244 (*AZD*), RSK inhibitor BI-D1870 (*BID*), and ROCK inhibitor Y27632 (*Y27*). *V*, empty vector control transfection; *trmt*, treatment.

We asked whether RSK regulates MLC phosphorylation in migratory epithelial cells. We stimulated A549 cells with PMA, which activates ROCK through PKC (66). ROCK induces MLC phosphorylation (14, 27). As expected, we observed a trend of increased phospho-MLC with PMA treatment, and this was abrogated by the ROCK inhibitor Y27632 (Fig. 5*A*, *Y27*). MEK and RSK inhibitors also reduced MLC phosphorylation, although not to the level of ROCK inhibition (Fig. 5*A*).

We sought to determine how Ser-507 phosphorylation impacts MYPT1 molecular function. Because myosin phosphatase activity is controlled by ROCK-mediated inhibitory phosphorylations, we tested whether MYPT1 Ser-507 phosphorylation regulates ROCK interaction with MYPT1. We co-transfected cells with HA-MYPT1 Ser-507 mutants and assessed co-immunoprecipitation with ROCK. We found that PMA reduced ROCK–MYPT1 interaction, indicating that RSK and ROCK activation disassociates the ROCK–MYPT1 complex (Fig. 5*B*, *WT*). Kinase–substrate dissociation upon stimulation have been observed in similar signaling scenarios, including ERK dissociation from RSK and PKC dissociation from peptide substrates (67, 68). MYPT1 S507A did not affect the interaction, suggesting that the WT interaction is at the limit of detection. MYPT1 S507D increased ROCK–MYPT1 association (Fig. 5*B*). These data suggest that RSK phosphorylation of MYPT1 Ser-507 may promote ROCK's interaction with and inhibition of MYPT1.

Because Thr-853 phosphorylation regulates MLCP interaction with myosin (30–32), we assayed the MYPT1 Ser-507 mutants' ability to bind myosin *in vitro*. We immunoprecipitated FLAG–PP1C/HA–MYPT1 complexes from 293T cells and incubated the complexes with purified full-length myosin. The MYPT1 S507A mutant bound myosin more than WT MYPT1, similar to the T853A mutant (Fig. 5*C*). In contrast, the S507D phosphomimetic mutant interacted with myosin at a similar level as WT MYPT1. Thus, MYPT1 Ser-507 phosphorylation inhibits interaction with myosin.

We next assayed whether MYPT1 Ser-507 phosphorylation regulates myosin activity in a collagen gel contraction assay. During growth and migration in 3D, myosin II–mediated contractile force remodels and compacts the extracellular matrix (14, 69). The 3658 cells contract collagen gels similar to fibroblasts (69) (Fig. 5*D*). We found that over the course of 48 h, 3658 cells expressing MYPT1 S507D contracted collagen gels significantly more than cells with WT MYPT1 (Fig. 5*D*, 39% ± 3% for S507D *versus* 22% ± 2% for WT, *p* = 0.03, Mann–Whitney *U* test). Together, these data show that MYPT1 Ser-507 phosphorylation is sufficient to inhibit MLCP activity and promote myosin II–mediated contractility.

**Figure 6.**
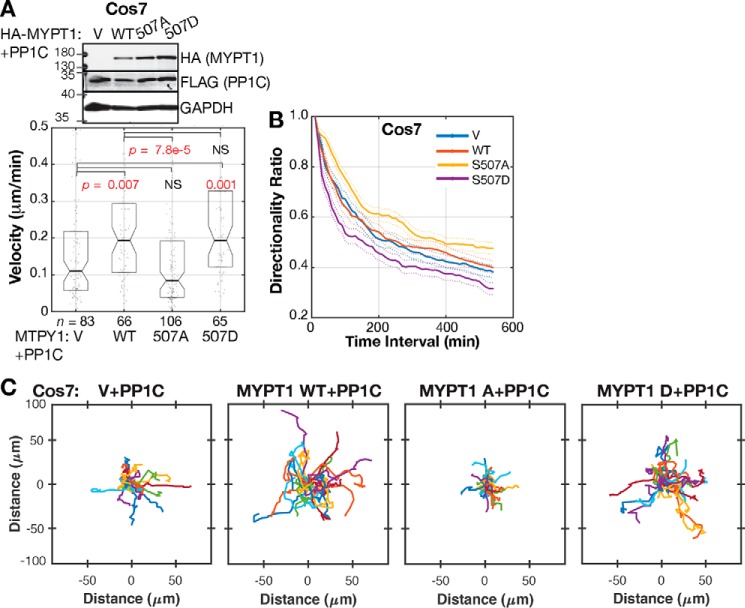
**RSK phosphorylation of MYPT1 promotes Cos7 cell migration.** *A*, Western blot and migration velocity of Cos7 cells co-overexpressing HA-MYPT1 and FLAG-PP1C and co-transfected with Emerald-Lifeact. *n* = number of Emerald-positive cells tracked from at least three independent experiments. *Boxes* span second and third quartiles (25^th^ to 75^th^ distribution). Notches are 95% CI around the median. *p* value from Kolmogorov–Smirnov test. Samples with distributions distinct from control have *p* values labeled in *red. B*, mean directionality of cells analyzed in *A*, plotted for each time interval. The *dashed lines* show S.E. *C*, plots of migrating Cos7 cells with HA-MYPT1 and FLAG-PP1C co-expression. Tracks of the 25 cells in *A* with migration speed closest to the median are depicted.

We tested whether RSK phosphorylation of MYPT1 Ser-507 is necessary for cell migration. We co-transfected Cos7 and HT1080 cells with HA-MYPT1 and FLAG-PP1C to maintain MLCP stoichiometry and Emerald-LifeAct to specifically label the transfected cells for migration tracking (Fig. 6*A* and Fig. S4*A*). Cells co-expressing MYPT1 with PP1C migrated faster than cells with only PP1C overexpressed (Fig. 6, *A*, *WT*, and *C*). However, cells co-expressing MYPT1 S507A and PP1C migrated significantly slower than cells with WT MYPT1. Median migration velocity of 0.194 μm/min in Cos7 cells with HA-MYPT1 WT and FLAG-PP1C was reduced to 0.084 μm/min in Cos7 cells with HA-S507A and FLAG-PP1C, *p* = 7.8E-5; Fig. 6, *A* and *C*). Similarly, median migration velocity of 0.319 μm/min in HT1080 cells with HA-MYPT1 WT and FLAG-PP1C was reduced to 0.217 μm/min in HT1080 with HA-S507A and FLAG-PP1C, *p* = 0.026, (Fig. S4). In both Cos7 and HT1080 cells, MYPT1 S507D expression was not significantly different from that of WT MYPT1, indicating that MYPT1 + PP1C co-overexpression promotes migration to an extent that cannot be further enhanced by MYPT1 Ser-507 phosphorylation.

The effects of MYPT1 Ser-507 mutation on migration directionality followed a similar pattern as found with the RSK inhibitors. Cos7 cells expressing the MYPT1 S507A mutant were more persistent, and cells expressing the S507D mutant were less persistent than cells expressing WT MYPT1 (Fig. 6*B*). In HT1080 cells, MYPT1 S507A expression also induced more persistent migration than WT MYPT1 (Fig. S4*C*). In contrast, but consistent with the lack of an effect on velocity, S507D expression in HT1080 cells did not significantly alter persistence beyond that of WT MYPT1 (Fig. S4*C*).

**Figure 7.**
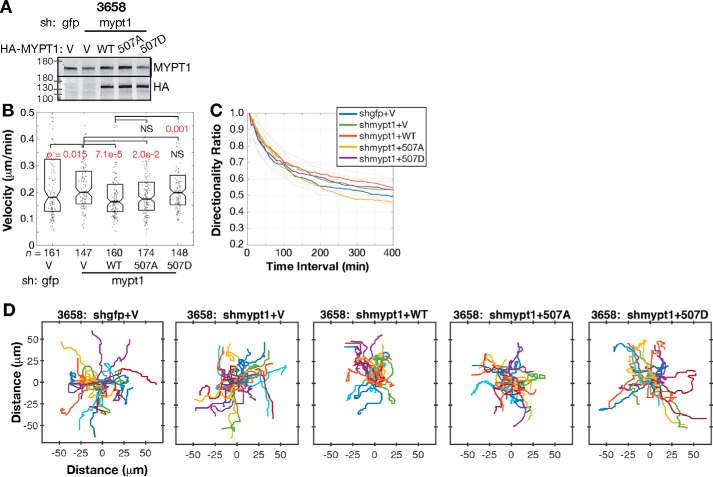
**MYPT1 Ser-507 phosphorylation promotes 3658 cell migration.** *A*, Western blot of murine 3658 cells with stable MYPT1 knockdown and replacement with human MYPT1 WT and Ser-507 mutants. *B*, migration velocity of *n* cells tracked from at least three independent experiments. *Boxes* span second and third quartiles (25^th^ to 75^th^ distribution). Notches are 95% CI around the median. Samples with distributions distinct from control have *p* values labeled in *red* (Kolmogorov–Smirnov test). *C*, average directionality of 3658 cells analyzed in *B*, plotted for each time interval. The *dashed lines* show S.E. *D*, plots of migrating 3658 cells with stable MYPT1 knockdown and replacement. Tracks of the 25 cells in *B* with migration speed closest to the median are depicted.

We also carried out similar migration assays in murine 3658 cells, in which we could stably knock down the endogenous murine MYPT1 and complement with human MYPT1. Partial shRNA knockdown was complemented by viral transduction of HA-MYPT1 constructs, expressed at nearly endogenous levels (Fig. 7*A*). Reduced MYPT1 levels increased migration (Fig. 7*A*, sh gfp+V *versus* sh mypt1+V *p* = 0.001). This suggests that low-level MLCP inhibition optimizes migration velocity. In this case, the 3658 cells with MYPT1 S507A migrated similar to those with WT MYPT1 (*p* = 0.001; Fig. 7, *B* and *D*), and cells with MYPT1 S507D migrated significantly faster than those with WT MYPT1 (*p* = 0.03; Fig. 7, *B* and *D*). 3658 cell migration persistence among the different MYPT1 Ser-507 conditions was indistinguishable (Fig. 7*C*). These migration assays in three distinct cell types using either stoichiometric MLCP overexpression or MYPT1 knockdown and replacement indicate that RSK phosphorylation of MYPT1 Ser-507 induces a moderate level of MLCP inhibition that promotes motility.

We next tested the relative effects of RSK and ROCK inputs into MLCP during cell migration. Our findings on RSK phosphorylation of MYPT1 Ser-507 suggest that ERK-activated RSK reduces MLCP activity (and thereby increases myosin activity and cell migration) by promoting ROCK's inhibitory phosphorylation of MYPT1 Thr-853. ROCK, however, has two mechanisms for MLCP inhibition: direct inhibitory phosphorylation of MYPT1 and also phosphorylation and activation of the CP1–17 inhibitory protein (70, 71). In response to thromboxane G protein–coupled receptor signaling, ROCK phosphorylates CPI-17 at Thr-38 and phospho-CPI-17 directly and selectively binds and inhibits MLCP (70, 71). We hypothesized that U46619 application would activate ERK/RSK and ROCK signaling to turn off the MLCP and induce Cos7 cell migration. In this case, manipulation of MYPT1 levels or the Ser-507 phosphosite would not impact migration in the presence of activated CPI-17. We co-transfected Cos7 cells with Vector or HA-MYPT1, along with FLAG-PP1C and H2B-mCherry to label and track the transfected cells. We treated the cells with U46619 while assaying migration (Fig. S5). Median migration velocity of cells expressing only PP1C and treated with U46619 (0.09 μm/min) was unchanged by HA-MYPT1 co-expression (0.10 μm/min, *p* = 0.1; Fig. S5). The S507A (0.13 μm/min) and S507D (0.11 μm/min) mutations also did not impact U46619-induced migration (*p* = 0.3 for WT *versus* S507A and 0.9 for WT *versus* S507D; Fig. S5, *A* and *C*). Persistence was also not regulated (Fig. S5*B*). Thus, U46619-mediated activation of both RSK and ROCK abrogates regulation by the MYPT1 Ser-507 phosphosite.

**Figure 8.**
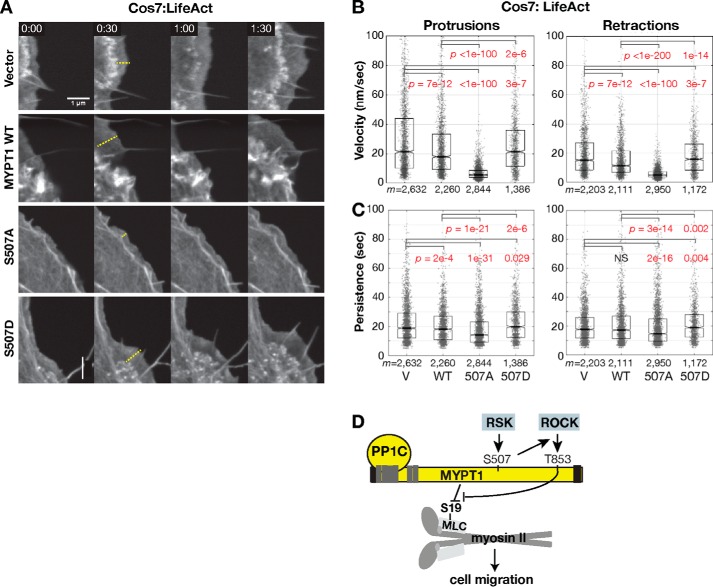
**RSK phosphorylation of MYPT1 promotes edge velocity.** *A*, representative images of migrating Cos7 cells co-transfected with HA-MYPT1, FLAG-PP1C, and Emerald-Lifeact. The *dashed lines* show the protrusive region. *B*, protrusion and retraction velocity distributions. *C*, protrusion and retraction persistence distributions of *m* significant protrusion events in *n* = 8 cells for vector (*V*), the WT sequence (*WT*), and S507A and *n* = 4 cell for S507D. *Boxes* span the 25^th^ to 75^th^ distribution. Each data point is the mean velocity for that event. The *central horizontal line* indicates the median for all events. Notches are 95% CI of median. Samples with distributions distinct from control have *p* values labeled in *red* (Kolmogorov–Smirnov test). *D*, model of MYPT1 regulation and action on myosin.

We tested whether MYPT1 Ser-507 phosphorylation-induced myosin II activity is involved in lamella-driven edge motion. We co-transfected Cos7 cells with HA-MYPT1, FLAG-PP1C, and Emerald-LifeAct and imaged steady-state dynamics (Movies S1–S4). The cells with empty vector or WT MYPT1 exhibited an area of low actin density that expanded behind the edge of treadmilling actin during protrusion (Fig. 8*A*, *Vector* and *MYPT1 WT*). MYPT1 S507A cells exhibited thin protrusions (Fig. 8*A*, *S507A*). MYPT1 S507D cells exhibited lamella expansion similar to WT cells (Fig. 8*A*, *S507D*).

We calculated edge protrusion and retraction dynamics and found that MYPT1 Ser-507 phosphorylation is necessary for protrusion and retraction velocity and minimally affects persistence (Fig. 8, *B* and *C*). Co-expressing MYPT1 and PP1C reduced mean protrusion velocity, as expected if increased phosphatase activity reduces lamella myosin activity (21.2 nm/s mean protrusion velocity in PP1C-only transfected cells reduced to 17.9 nm/s with MYPT1 co-transfection, *p* < 0.05, Kolmogorov–Smirnov test; Fig. 8*B*). The MYPT1 S507A mutant reduced mean protrusion velocity beyond that of WT MYPT1 (mean protrusion velocity, 5.6 nm/s; *p* < 0.05, Kolmogorov–Smirnov test; Fig. 8*B*). In contrast, cells expressing the S507D mutant exhibited protrusion dynamics similar to vector-transfected cells (mean protrusion velocity, 21.3 nm/s; Fig. 8*B*). A similar pattern of regulation was observed in retractions and in the 25% fastest protrusion and retraction events (Fig. S6). Retraction velocity was reduced with MYPT1 S507A expression and increased with S507D, compared with WT MYPT1 expression.

Similar to treatment with the MEK and RSK inhibitors, MYPT1 Ser-507 mutation nominally altered protrusion and retraction persistence. Mean protrusion duration of 18.1 s in WT MYPT1-expressing cells was reduced to 14.2 s with S507A expression and increased to 19.8 s with S507D expression (*p* < 0.05, Kolmogorov–Smirnov test; Fig. 8*C*). Retraction persistence of 17.2 s in WT MYPT1-expressing cells was reduced to 14.7 s with S507A expression and increased to 18.9 with S507D expression (*p* < 0.05, Kolmogorov–Smirnov test; Fig. 8*C*). Thus, RSK signaling to MYPT1 Ser-507 promotes lamella expansion and edge motion for cell migration.

These data point to a model (Fig. 8*D*) in which RSK phosphorylation of MYPT1 Ser-507 promotes ROCK interaction with MYPT1 and inhibition of MLCP interaction with myosin, thereby increasing MLC phosphorylation and myosin activity for cell edge motion and migration. Because other RSK substrates involved in the cytoskeletal dynamics of cell migration have been identified (59, 72–75), we tested the role of the RSK–MYPT1 signal in RSK-mediated edge dynamics. We co-transfected Cos7 cells with MYPT1 and PP1C and assayed edge protrusion under RSK inhibition. We used a slower (5 s) sampling rate for image acquisition than in previous experiments (Fig. 4), which removed some of the high frequency events and resulted in slower calculated edge motion (Fig. S7). Cells treated with DMSO exhibited the same pattern as previously observed, in which co-expressing MYPT1 and PP1C reduced mean protrusion velocity (11.5 nm/s mean protrusion velocity in vector-transfected cells reduced to 10.0 nm/s with MYPT1 co-transfection, *p* < 0.05, Kolmogorov–Smirnov test; Fig. S7*A*). The MYPT1 S507A mutant reduced mean protrusion velocity beyond that of WT MYPT1 (mean protrusion velocity, 6.1 nm/s; *p* < 0.05), whereas the S507D mutant exhibited protrusion dynamics similar to vector-transfected cells (11.6 nm/s, *p* < 0.05; Fig. S7*A*). Under RSK inhibition with LJH685, a reduced protrusion velocity of 9.9 nm/s was further reduced to 8.6 nm/s with MYPT1 co-transfection (*p* < 0.05, Kolmogorov–Smirnov test; Fig. S7*C*). This indicates that myosin phosphatase is downstream of RSK signaling. However, we found that RSK inhibition largely masked the effects of MYPT1 mutation. The MYPT1 S507A mutant protrusion velocity did not differ from that of WT MYPT1 (mean protrusion velocity, 8.7 nm/s; *p* = 0.06), whereas the S507D mutant exhibited even slower protrusion dynamics (6.33 nm/s, *p* < 0.05; Fig. S7*C*). Retraction velocity was similarly regulated (Fig. S7*C*). Thus, as anticipated, RSK has additional cytoskeleton targets beyond MYPT1 that control edge dynamics.

## Discussion

We have uncovered a key molecular signal by which ERK/RSK regulates myosin II and cell edge dynamics during cell migration. Through the use of multiple agonists, inhibitors targeting multiple steps of the ERK/RSK pathway, and overexpression and knockout, we show that RSK1 and RSK2 are the major kinases responsible for phosphorylation of MYPT1 Ser-507. MYPT1 Ser-507 phosphorylation promotes ROCK interaction with MYPT1 and inhibits MLCP activity. This signal increases myosin activity. The reduction in cell migration with multiple RSK inhibitors and with the Ser-507 phospho-dead mutant indicates that this level of myosin phosphatase inhibition and myosin activation promotes epithelial cell migration. Further, it specifically drives edge protrusion and retraction velocities, which are dependent on actomyosin and focal adhesion dynamics.

Growth factor-mediated regulation of MYPT1 Ser-507 is primarily mediated by the RSKs in the panel of cell lines tested here (293T, 293E, Cos7, MDCK, A549, 3658, and HT1080). RSK1 and RSK2 knockout in 293T cells demonstrates that these two kinases are essential contributors to MYPT1 Ser-507 phosphorylation. Other AGC kinases that commonly phosphorylate similar motifs (43, 44) do not appear to target MYPT1 in these cells, because their agonists and inhibitors do not affect MYPT1 Ser-507 phosphorylation. This finding is consistent with the RSK kinases having a less-stringent phospho-motif compared with AKT and S6K (45, 76). However, insulin has been found to induce MYPT1 Ser(P)-507 in cells overexpressing insulin receptor and myoblasts (46, 47), suggesting that AKT and/or S6K may function as a third MYPT1 Ser-507 kinase in systems poised for insulin signaling to phosphatidylinositol 3-kinase/AKT/mTORC1/S6K. Indeed, in some cases MEK inhibition more completely abrogated Ser-507 phosphorylation than RSK inhibition (Fig. 1, *A* and *B*), leaving open the possibility of a MEK–ERK–mTORC1–S6K signal to MYPT1. Current RSK1 and RSK2 knockout data (Fig. 2*E*) do not distinguish between the residual MYPT1 Ser-507 phosphorylation being due to the remaining RSK1 or RSK2 homolog or the existence of a third MYPT1 Ser-507 kinase.

RSK's role in cell migration is complicated by reports of RSK1 and RSK2 having distinct function in different cell types (58–62). Most cells express RSK1 and 2, which are both targeted by the MEK and RSK inhibitors (63, 77, 78). Because these multiple MEK and RSK inhibitors reduce the migration of multiple cell lines, we conclude that overall RSK activity promotes cell motility.

We found that RSK activity and MYPT1 Ser-507 phosphorylation are necessary for optimal edge velocity and cell motility. ERK and RSK inhibitors and MYPT1 S507A mutants inhibited edge protrusion, edge retraction, and cell migration velocities. In some cell types (Cos7, MDCK, 3658), RSK inhibitors and MYPT1 S507A expression also promoted migration persistence. As ROCK inhibition increases persistence (35, 37), these results are consistent with a model in which phospho-MYPT1 Ser-507 promotes ROCK-mediated inhibition of the phosphatase. The cell motility parameters and ROCK-myosin II signal levels that distinguish these persistence responses remain to be determined.

Myosin II contracts actin fibers coupled to focal adhesions in the lamella, generating tension that balances protrusive forces against the membrane and prevents nonproductive edge ruffling (4–7, 79). Myosin II can also induce adhesion maturation and stress fiber formation, which slow migration (80). Moderate induction of myosin II activity, such as low expression of constitutively active MLC (MLC-DD), low-level MLCP inhibition, and extracellular matrix manipulation, optimizes migration velocity (35, 81–83). However, excessive tension induced by high MLC-DD expression reduces membrane protrusion and migration velocity (35, 83). Thus, a graded mechanism for inducing myosin activity is needed to tune cell migration to environmental cues. Consistent with this, the S507D phosphomimetic mutant increases ROCK interaction with and inhibition of MYPT1. The S507D-induced myosin activity does not cause overall edge retraction but rather increases edge retraction velocity to enable productive cell migration. In this manner, growth factor or oncogenic mutation-induced increases in ERK/RSK signaling can moderately induce myosin II activity to a level that promotes, rather than inhibits, cell motility.

Additional ERK-mediated, RSK-dependent, and RSK-independent mechanisms for myosin II regulation appear to contribute to the overall loss of cell migration and edge motion with the MEK and RSK inhibitors. A recently identified RSK–LARG–ROCK pathway suggests that in glioma cells, RSK may also inhibit MYPT1 and promote motility through phosphorylation and activation of the LARG RhoGEF, which increases RhoA activation (84). RSK2 can also transcriptionally up-regulate fascin, the main actin bundler in microspikes (74, 85). Myosin II associates with the base of actin microspikes (85), which grow into filopodia that integrate into the lamella's contractile actin array that integrates with adhesions (9–11). In this manner, RSK2-induced fascin may participate in edge stabilization during protrusion and myosin II-dependent edge retraction. RSK2 has also been found to promote optimal migration by balancing recruitment of the actin cross-linker filamin to adhesions, which would increase tension and adhesion maturation, with integrin inactivation (58). Further, we have previously identified an ERK-mediated RSK-independent connection to myosin II. ERK promotes actin polymerization and lamella expansion via activation of the WAVE complex (1, 2, 20, 21). Actin polymerization against the cell membrane increases cortical tension, which induces mechanosignals to further activate the myosin II within focal adhesions (1, 2, 7, 21). Such an indirect mechanism for ERK regulation of myosin activity likely contributes the observed edge motion phenotypes with MEK inhibitor.

In some homeostatic biological contexts, increased ERK signaling to myosin II appears to have little consequence, whereas in others, increased RSK–MYPT1 signaling enables phenotypic proficiency. For example, the MYPT1 S507D phosphomimetic mutation promoted edge motion and migration to a similar level as WT MYPT1 when overexpressed, suggesting that the level of MYPT1 Ser-507 phosphorylation in overexpressed WT MYPT1 is optimal for cell migration. These phenotypes were different when endogenous MYPT1 was knocked down before assaying migration and when myosin activity was assayed by collagen gel contraction. In these latter cases, cells expressing MYPT1 S507A migrated and contracted the gel similar to cells with WT MYPT1, whereas cells with MYPT1 S507D exhibited increased migration and contractility. Thus, increased inhibition of MYPT1 and activation of myosin II promoted cell migration and gel contraction.

In conclusion, we have discovered a signaling mechanism by which ERK controls myosin II and lamella function. By controlling both actin polymerization (20, 21) and myosin II–mediated contractility, the RAS/ERK pathway can coordinately promote and integrate multiple steps of cell motility. The ERK-activated RSK phosphorylates MYPT1 Ser-507, which induces inhibition of the myosin phosphatase by ROCK. This signal promotes a level of myosin II activity optimal for protrusion and retraction cycles during motility. We expect the RSK–MYPT1 signaling paradigm plays a role in migration-driven physiology, especially the invasion of cancers with activated ERK (86) and convergence during gastrulation, which requires both ERK and ROCK–MYPT1 activity (37, 87). Future work is needed to determine the role of ERK/RSK signaling and RSK control of MLCP in other myosin II-driven cellular processes, such as tension at cell–cell contacts in epithelial monolayers (88, 89).

## Experimental procedures

### Reagents and plasmids

The cells were treated with 40 ng/ml PMA, 50 ng/ml EGF, 100 nm insulin, 10 μg/ml anisomycin, or 5 μm U46619. Inhibitors were used at the following concentrations: AZD6244, 5 μm; U0126, 20 μm; SCH772984, 2.5 μm; BI-D1870, 5 μm; LJH685, 5 μm; Y27632, 10 μm; SB203580, 10 μm; rapamycin, 20 nm; AKT VIII, 10 μm; PF-4708671, 10 μm; and staurosporine, 1 μm. Human MYPT1 cDNA from Open Biosystems was cloned into pRK7 and pBabeNeo with N-terminal HA tags. pECE-M2-PP1Cβ (90) and pmEmerald-LifeAct were gifts from Anne Brunnet and Michael Davidson (Addgene catalog no. 31677 and 54148). Human RSK1–4 were cloned into pcDNA.3 with N-terminal HA. CRISPR oligonucleotides were cloned into pSpCas9(BB)-2A-Puro (Addgene catalog no. 62988, cloned by UofU HSC Mutation Generation and Detection Core). Sequences were as follows: RSK1-28, CACCGTCTCCATCTTGGTCCGGACG; RSK1-37, CACCGTTTGCAGGTGATGTTCACGG; RSK2-65, CACCGCAGGAAGAAGGCGTCGTGA; RSK2-70, CACCGGACCGAGTGAGATCGAAGA; RSK3-40, CACCGAGCCCGTCCGACAGCGCTG; and RSK3-45, CACCGGAACGTGATATCTTGGTAG. Mouse MYPT1 shRNA was single Sigma TRCN0000240623, selected for its targeting the 3′-UTR and effective knockdown.

### Cell culture

3658 cells (56) were a gift from Eric Snyder. All other lines were from ATCC. All were cultured in DMEM, 10% FBS. The cells were transfected with Lipofectamine 2000 and TransIT-293, TransIT-X2 (HT1080), or TransIT-2020 (Cos7 and 3658). 16–20 h after transfection, the cells were starved in DMEM, 0% FBS containing 20 mm HEPES, for 24 h. CRISPR knockouts were generated by Cas9/CRISPR transfection, 2-day puromycin selection, dilution cloning, and sequencing. Stable cell populations were selected with 2–2.5 μg/ml puromycin or 750 μg/ml G418.

### Real-time PCR

RSK3-specific antibodies were unreliable, so RT-PCR was performed to validate the RSK3 knockout lines. Total RNA of cultured cells was extracted using TRIzol with the PureLink RNA mini kit. cDNA was synthesized from 250 ng of extracted RNA using the iScript reverse transcription Supermix (Bio-Rad). Real-time quantitative PCR was performed in four replicates with the SsoAdvanced Universal Probes Supermix (Bio-Rad) and detected with a Bio-Rad CFX Connect Real-Time PCR detection system. Primers and probes for RPS6KA2 (RSK3) and GAPDH were TaqMan Hs00179731_m1 and Hs00266705_g1. Relative RSK3 transcript levels were determined by calculating 2^−ΔΔCt^ values normalized to GAPDH. Similar results were obtained in three biological replicates.

### Cell lysis for Western blots and endogenous MYPT1 IPs

After stimulation, the cells were washed once with cold PBS and then lysed in radioimmune precipitation assay buffer (10 mm Tris-Cl, pH 8.0, 1 mm EDTA, 0.5 mm EGTA, 1% Triton X-100, 0.1% sodium deoxycholate, 0.1% SDS) with 1 mm sodium orthovanadate, 1 mm phenylmethylsulfonyl fluoride, 5 μg/ml leupeptin, 5 μg/ml aprotinin, and 5 μg/ml pepstatin A. The cell extracts were centrifuged at 14,000 rpm for 10 min to remove cell debris, and the cellular supernatant was quantified and normalized using a Bradford or BCA assay. Cell lysates and immunoprecipitates using CST anti-MYPT1 2634 were subjected to SDS-PAGE and transferred to nitrocellulose membranes.

### Antibodies

Primary antibodies were sourced as follows: p-MYPT1 Ser-507, antibody 3040; p-MYPT1 Ser-668, antibody 3048; p-MYPT1 Thr-853, antibody 4563; p-MYPT1 Thr-696, antibody 5163; MYPT1, antibody 2634; p-RSK T359/S363, antibody 9344; p-RSK Thr-359, antibody 8753; p-RSK Ser-380, antibody 9341; pan-RSK, antibody 9355; RSK1, antibody 8408; RSK2, antibody 5528; p-AKT Ser-473, antibody 4060; AKT, antibody 9272; S6K, antibody 9202; p-S6 235/236, antibody 4858; S6, antibody 2317; p-p38, antibody 9211; ERK, antibody 9102 (CST); FLAG M5 and p-ERK, antibody M9692 (Sigma); HA.11 (Covance), ROCK1 antibody A300-455 and A300-457 (Bethyl); MLC, antibody ab92721; and p-MLC S20, antibody ab2480 (Abcam). 680LT and 800CW IRDye-labeled secondary antibodies were from Li-COR or Fisher. Membranes for phospho-MYPT1, MYPT1, and myosin antibodies were first blocked with 5% (w/v) BSA in TBS (10 mm Tris, pH 7.4, and 150 mm NaCl), followed by primary antibody incubation in a 1:2 mixture of Odyssey blocking buffer and TBS. All others were blocked with 5% nonfat dry milk in TBS. After incubation with primary and secondary antibodies, the membranes were washed with TBS-T (TBS with 0.1% Tween 20). Western blots were imaged on a Li-COR Odyssey Imager. Marker lanes visible in the 680 channel scans were overlaid onto the 800 channel scans.

### Western blotting quantification

Intensity values were quantified in ImageStudio (LI-COR). Generally, phospho-signals were normalized to the total protein level of the same protein. In cases in which this introduced an unacceptable level of uncertainty by requiring incomplete stripping of a phospho-signal before carrying out Western blotting for the total protein or requiring the samples to be re-run on an independent gel, normalization was carried out to total ERK. Total ERK levels do not change with the short-term stimulations and inhibitor treatments in this work (see total ERK levels in Figs. 1, 2, 4, and S1). Standard deviation between experimental replicates was calculated and graphed in MATLAB.

### ROCK–MYPT1 co-immunoprecipitation

Cells were lysed in 20 mm Tris, pH 7.5, 0.5% Nonidet P-40, 125 mm NaCl. For the IP, 2 mg of cell lysate was diluted in the same buffer but with reduced NP-40 content for a final concentration of 0.25% NP-40. The lysates were precleared by rotating incubation with rabbit IgG and protein A (4 °C, 30 min), with a 2.4 × *g* centrifugation. Lysates were then incubated, rotating with a 1:1 ratio of Bethyl anti-ROCK1 A300-455 and A300-457 antibodies, which target distinct ROCK epitopes for an hour at 4 °C. A 1:1 mixture of protein A– and protein G–Sepharose beads were added, and the mixtures were rotated for an additional 30 min at 4 °C. The beads were washed three times with 20 mm Tris, pH 7.5, 0.15% Nonidet P-40, 125 mm NaCl, using centrifugation of 2.4 × *g*. Immunoprecipitates were subjected to SDS-PAGE and analyzed by immunoblotting.

### Myosin-binding assay

293T cells were co-transfected with HA-MYPT1 and FLAG-PP1C at 2:1 ratio, stimulated with 40 ng/ml PMA, washed once with PBS, and Dounce homogenized in lysis buffer: 0.3 m NaCl, 20 mm Tris-HCl, pH 8.0, 0.1 mm EGTA, 0.1 mm EDTA, 2 mm DTT, 5% glycerol, 0.1% Tween 20, and protease and phosphatase inhibitors (Thermo Fisher Halt mixture, pepstatin A, and phenylmethylsulfonyl fluoride). The lysates were clarified by centrifugation at 15,000 rpm for 15 min (4 °C). Following immunoprecipitation with anti-FLAG M2 affinity gel (Sigma), complexes were washed twice with extraction buffer and twice with binding assay buffer: 10 mm HEPES, pH 7.5, 50 mm KCl, 5 mm MgCl_2_, and 2 mm DTT. Full-length bovine cardiac muscle myosin (Cytoskeleton) was incubated with the bound MYPT1/PP1C for 20 min at 30 °C, washed three times with binding buffer, and assessed for interaction by SDS-PAGE and Western blotting.

### Collagen contraction assay

The cells were embedded in 1 mg/ml neutralized rat tail collagen I. Gel images were taken with a Bio-Rad Gel Doc System and gel quantified in Fiji. Each time point was calculated as an average of triplicate wells from three or four independent experiments.

### Migration assays

The cells were plated on acid-treated glass. Two days after plating and just before imaging, the medium was exchanged for fresh DMEM, 10% FBS with 20 mm HEPES. For Cos7 and HT1080 migration assays involving identification of Lifeact-GFP or H2B-mCherry positive cells, the medium was changed to Fluorobrite media (Invitrogen), 10% FBS, 20 mm HEPES. Cell migration was imaged by phase contrast microscopy, at 37 °C, 5% CO_2_ on a Nikon Ti inverted microscope with a Plan Fluor ELWD 20× air objective and an environmental chamber. Images were acquired with an Andor Clara CCD camera using Metamorph or Elements. The cells were imaged every 5 (3658) or 10 min (MDCK, HT1080, A549, Cos7) and manually tracked for ∼4 h (MDCK, HT1080), 6–7.5 h (3658), or 8 h (Cos7 and A549) using Fiji “Trackpoints.” The data were analyzed in MATLAB. Velocity was calculated as distance/time. The two-sample nonparametric Kolmogorov–Smirnov test at 5% significance was used to determine whether the population distributions differ significantly. Cell motion was tested for characteristics of persistent random walk, in which the mean squared displacement increases in a superdiffusive manner: MSD(t) ∝ t^α^, where 1<α<2). Consistent with previous work in MDCK cells (91), ∼50% of the cells in each population met the assumptions of a persistent random walk. Thus, directionality was calculated as an average of the straight-line distance between the trajectory's start point and current position for time *t*, divided by the actual trajectory length, for a population of cells at each given time point. The continuous, one-dimensional probability distribution of each inhibitor or point-mutant sample was compared with the corresponding control DMSO or WT probability distribution.

### Cell edge analysis

Cells expressing Emerald-LifeAct or F-tractin were plated in Fluorobrite (Invitrogen), 10% FBS, 20 mm HEPES on acid-treated glass and imaged on a Nikon Ti inverted microscope with a CFI Apo TIRF 60× oil, 1.45 NA objective using Perfect Focus, Yokagawa CSU-10 spinning disk confocal and Spectral Applied Research Borealis modification, 488 solid-state laser, and Photometrics Myo CCD camera with Metamorph. The cells were imaged every 5–10 s for 10 min, with 300–500-ms exposures. Displayed images were scaled to the same grayscale range (90–900). Post-image edge analysis was carried-out in MATLAB as described previously (20, 21). Population distributions were tested for equality using a two-sample nonparametric Kolmogorov–Smirnov test at 5% significance. The continuous, one-dimensional probability distribution of each inhibitor or point-mutant sample was compared with the corresponding control DMSO or WT probability distribution.

## Author contributions

S. C. S., A. E., B. D. M., Y. K., J. P. B., and M. C. M. data curation; S. C. S., A. E., B. D. M., Y. K., K. R. C., and M. C. M. formal analysis; S. C. S., A. E., B. D. M., Y. K., and M. C. M. validation; S. C. S., A. E., and M. C. M. methodology; S. C. S., A. E., B. D. M., K. R. C., J. P. B., and M. C. M. writing-review and editing; J.B. and M. C. M. conceptualization; J. B. and M. C. M. supervision; J. B. and M. C. M. funding acquisition; M. C. M. writing-original draft.

## Supplementary Material

Supporting Information

## Footnotes

4 Please note that the JBC is not responsible for the long-term archiving and maintenance of this site or any other third party hosted site.

## Footnotes

3 The abbreviations used are:

ERK: extracellular signal-regulated kinase
MAPK: mitogen-activated protein kinase
MEK: MAPK/ERK kinase
RSK: p90 ribosomal S6 kinase
ROCK: Rho kinase
MYPT1: myosin phosphatase–targeting subunit 1
MLC: myosin light chain
MYLK: myosin light chain kinase
MLCP: myosin light chain phosphatase
MSK: mitogen- and stress-activated protein kinase
EGF: epidermal growth factor
ANOVA: analysis of variance
PMA: phorbol 12-myristate 13-acetate
PKC: protein kinase C
MDCK: Madin–Darby canine kidney
FBS: fetal bovine serum
GAPDH: glyceraldehyde-3-phosphate dehydrogenase
IP: immunoprecipitation
CI: confidence interval
RAF: rapidly accelerated fibrosarcoma.
