# Supplementary material for: p90 ribosomal S6 kinase (RSK) phosphorylates myosin phosphatase and thereby controls edge dynamics during cell migration
Source: J Biol Chem. 2019 May 28;294(28):10846–62. doi: 10.1074/jbc.RA119.007431 (PMC6635457; doi:10.1074/jbc.RA119.007431)
Supplement: Supporting Information [file supp_294_28_10846__index.html]

p90 ribosomal S6 kinase (RSK) phosphorylates myosin phosphatase and thereby controls edge dynamics during cell migration — RSK regulation of myosin phosphatase — Supporting Information 

# p90 ribosomal S6 kinase (RSK) phosphorylates myosin phosphatase and thereby controls edge dynamics during cell migration

## Supporting Information

- Supporting Information (to be published online) - Supporting Information - Figures S1-S7
- Supporting Information - Movie 1 - Supporting Information - Movie 1
- Supporting Information - Movie 2 - Supporting Information - Movie 2
- Supporting Information - Movie 3 - Supporting Information - Movie 3
- Supporting Information - Movie 4 - Supporting Information - Movie 4
