# Supplementary material for: p90 ribosomal S6 kinase (RSK) phosphorylates myosin phosphatase and thereby controls edge dynamics during cell migration
Source: J Biol Chem. 2019 May 28;294(28):10846–62. doi: 10.1074/jbc.RA119.007431 (PMC6635457; doi:10.1074/jbc.RA119.007431)
Supplement: Supporting Information [file supp_RA119.007431_142971_1_supp_332831_prmtqt.pdf]

**p90 ribosomal S6 kinase (RSK) phosphorylates myosin phosphatase and thereby controls edge dynamics during cell migration**

Shiela C. Samson, Andrew Elliott, Brian D. Mueller, Yung Kim, Keith R. Carney, Jared P. Bergman, John Blenis, and Michelle C. Mendoza

**Supporting Information**

1. **Figure S1**, RSK phosphorylates MYPT1 Ser-507
2. **Figure S2**, RSK controls cell migration speed and persistence in multiple cell types
3. **Figure S3**, RSK controls edge dynamics during cell motility
4. **Figure S4,** MYPT1 Ser-507 phosphorylation promotes HT1080 cell migration
5. **Figure S5,** MYPT1 RSK and ROCK co-activation negates migration regulation by MYPT1 Ser-507
6. **Figure S6**, MYPT1 Ser-507 phosphorylation promotes edge dynamics in Cos7 cells
7. **Figure S7,** RSK controls edge dynamics through additional MYPT1-independent mechanisms
8. **Movie 1.** Steady-state protrusion dynamics of Cos7 cells co-transfected with pRK7 vector, FLAG-PP1C, and Emerald-LifeAct.
9. **Movie 2.** Steady-state protrusion dynamics of Cos7 cells co-transfected with HA-MYPT1, FLAG-PP1C, and Emerald-LifeAct.
10. **Movie 3.** Steady-state protrusion dynamics of Cos7 cells co-transfected with HA-MYPT1 S507A, FLAG-PP1C, and Emerald-LifeAct.
11. **Movie 4.** Steady-state protrusion dynamics of Cos7 cells co-transfected with HA-MYPT1 S507D, FLAG-PP1C, and Emerald-LifeAct.

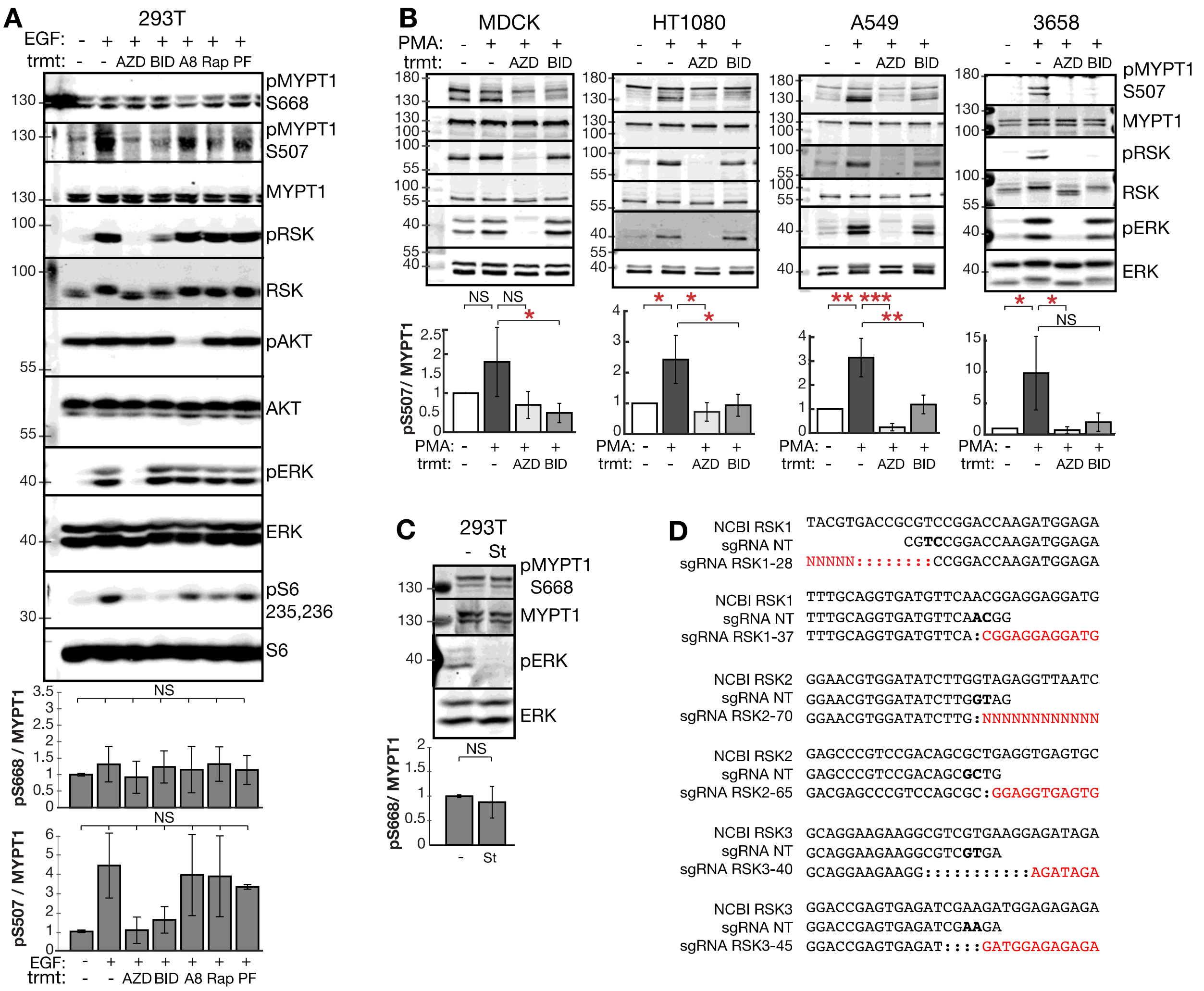


**Figure S1, RSK phosphorylates MYPT1 Ser-507. A.** Western and quantification of endogenous MYPT1 phosphorylation (pMYPT1 S507 and pMYPT1 S668) in 293T cells. pMYPT1 S507/total MYPT1 and pMYPT1 S668/total MYPT1 are relative to the signal in the starved condition without stimulation (normalized to 1.0). *n*=3 biological replicates. **B.** Western and quantification of endogenous MYPT1 Ser507 phosphorylation in MDCK, HT1080, A549, and 3658 cells (*n*=3 for each). **C.** Western and quantification of pMYPT1 S668 signal upon 5 min staurosporine (St) treatment. *n*=3 biological replicates. Error bars for Western quantifications are SD. Pathway agonists are: EGF (Epidermal Growth Factor) and PMA (phorbol 12-myristate 13-acetate). Inhibitors are: MEK inhibitor AZD (AZD6244), RSK inhibitor BID (BI-D1870), AKT inhibitor A8 (AKT VIII), mTORC1 inhibitor Rap (rapamycin), and S6 kinase inhibitor PF (PF-4708671). pRSK is pRSK T359, S363. pAKT is pAKT S473. pS6 is pS6 235,236. **D.** Sequences of RSK family CRISPR knockout clones. sgRNA NT is a CRISPR designed to not target human sequence. Red indicates mutant and frame-shifted sequence. sgRNA1-28 is antisense. All other sgRNAs are sense strand. RSK1-28 and RSK2-70 are biallelic heterozygotes. All other clones are biallelic homozygote mutants. One-way ANOVA * for $p<0.05$, ** for $p<0.01$, *** for $p<0.001$, and NS for $p>0.05$.

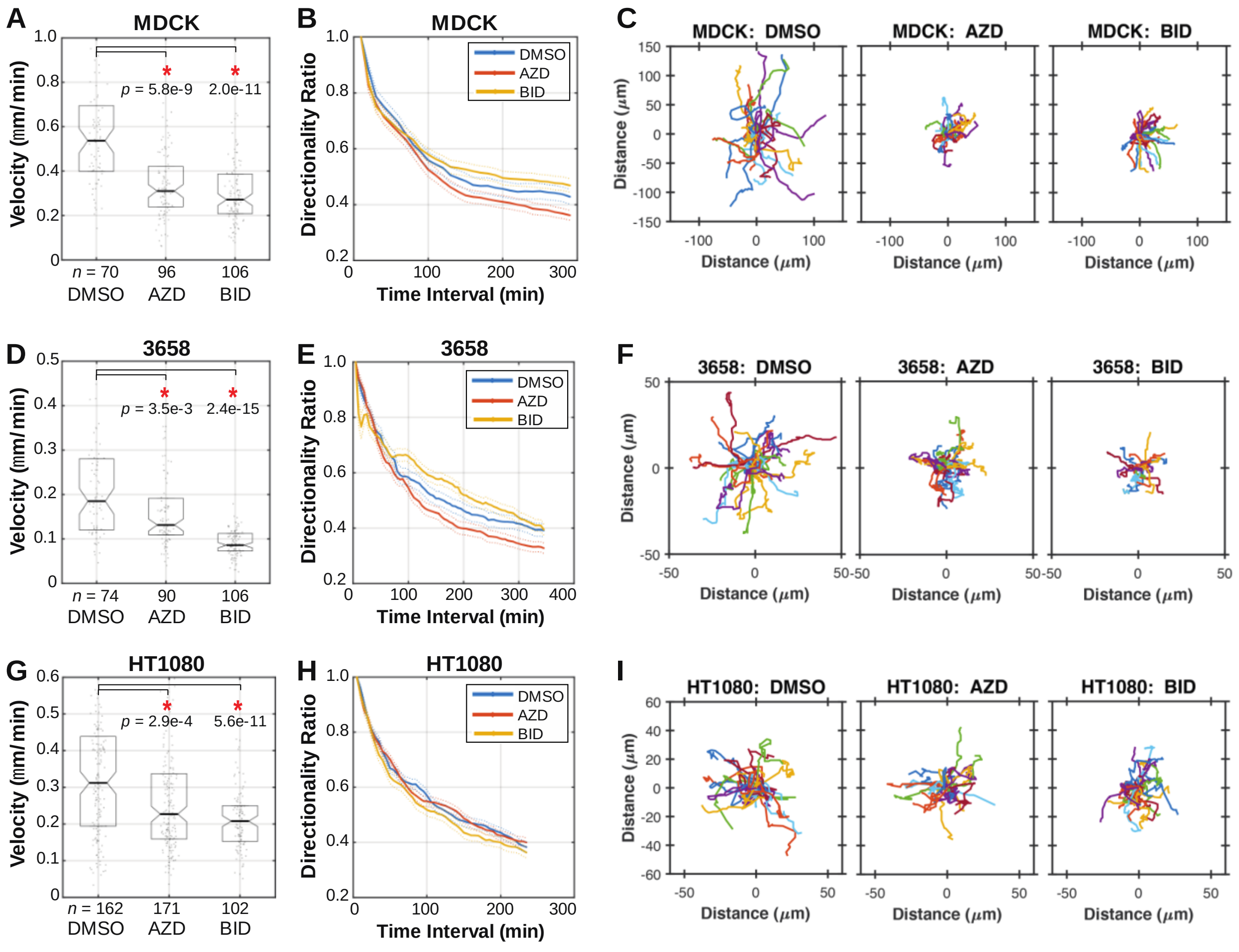


**Figure S2, RSK controls cell migration speed and persistence in multiple cell types. A., D., and G.** MDCK, 3658, and HT1080 cell migration velocity distributions of *n* cells tracked from at least 3 independent experiments. Boxes span the 25th to 75th distribution. Central horizontal line indicates the median. Notches are 95% C.I. around the median. Asterisk shows samples with distributions distinct from control DMSO treatment, Kolmogorov-Smirnov test. **B.**, **E.**, and **H.** Average directionality of cells analyzed in A., D., and G., plotted for each time interval. Dashed lines show SEM. **C.**, **F.**, and **I.** Plots of migrating MDCK, 3658, and HT1080 cells treated with DMSO, MEK inhibitor AZD6244 (AZD) or RSK inhibitor BI-D1870 (BID). Tracks of the 25 cells with migration speed closest to the median are depicted.

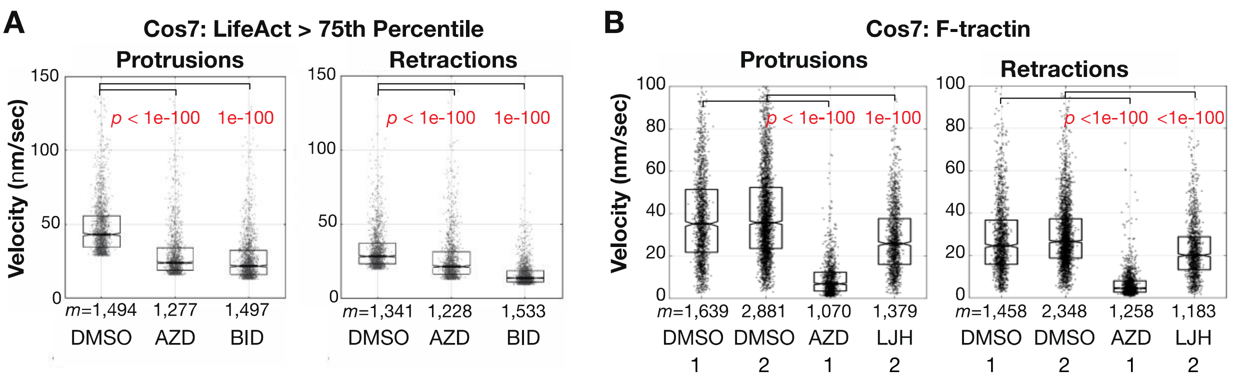


**Figure S3, RSK controls edge dynamics during cell motility. A.** Protrusion and retraction velocity distributions of the top 25% of events in the corresponding Cos7 cells expressing LifeAct in Figure 4B. *n* = 6 cells treated with DMSO, 5 cells with AZD6244, and 6 cells with BI-D1870. **B.** Mean protrusion and retraction velocity distributions of Cos7 cells expressing F-tractin. DMSO1 and AZD1 were imaged on the same day. DMSO2 and LJH2 were imaged on a distinct day. *n* = 2 cells for each condition. Boxes span 2nd and 3rd quartiles (25$^{th}$ to 75$^{th}$ distribution). Notches are 95% C.I. around the median. *P* values in red for samples with distributions distinct from control DMSO treatment, Kolmogorov-Smirnov test.

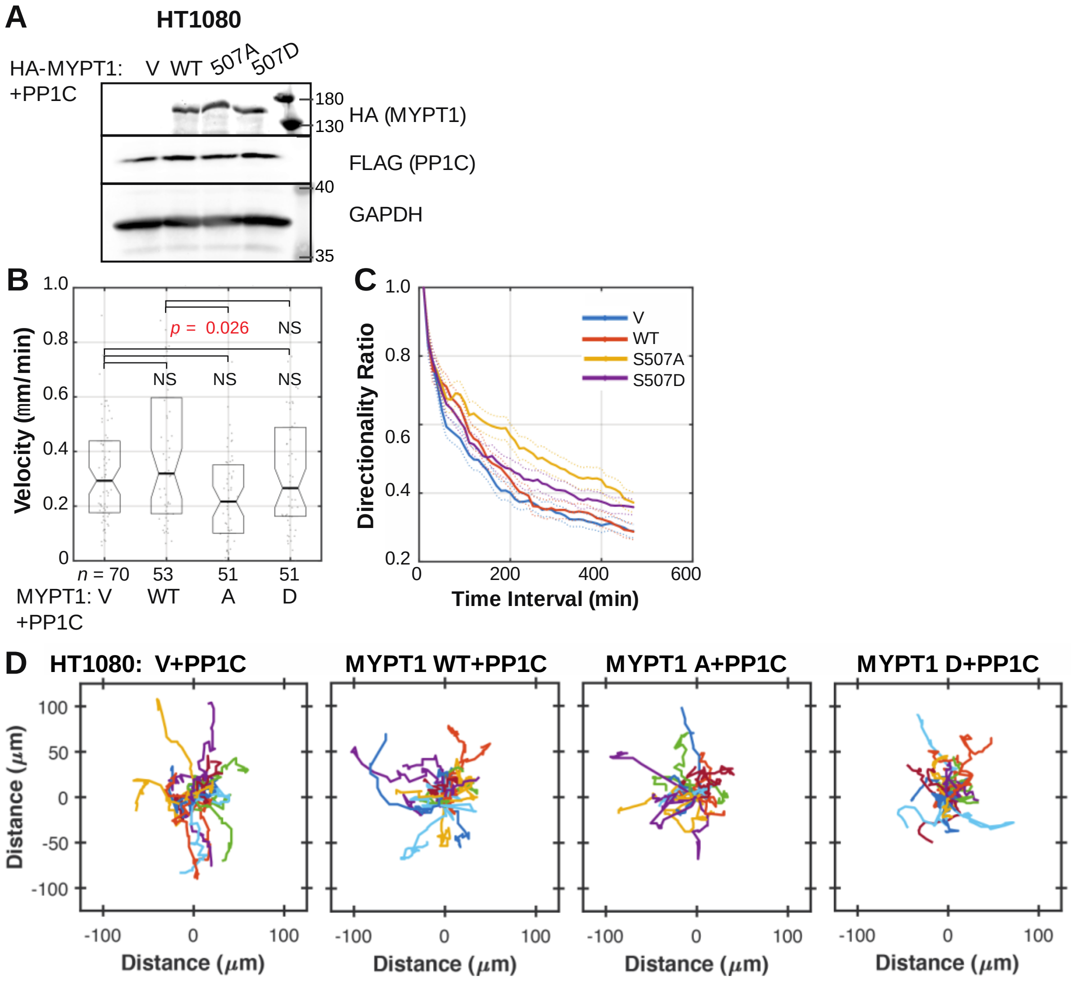


**Figure S4, MYPT1 Ser-507 phosphorylation promotes HT1080 cell migration. A.** Western of HT1080 cells with HA-MYPT1 and FLAG-PP1C co-expression. **B**. Cell migration velocity distributions of *n* transfected HT1080 cells tracked from at least 3 independent experiments. Boxes span the 25$^{th}$ to 75$^{th}$ distribution. Central horizontal line indicates the median. Notches are 95% C.I. around the median. Samples with distributions distinct from control have *p* values labeled in red, Kolmogorov-Smirnov test. **C.** Average directionality of cells analyzed in B., plotted for each time interval. Dashed lines show SEM. **D.** Plots of migrating HT1080 transfected with HA-MYPT1 and FLAG-PP1C. Tracks of the 25 cells with migration speed closest to the median in B are depicted.

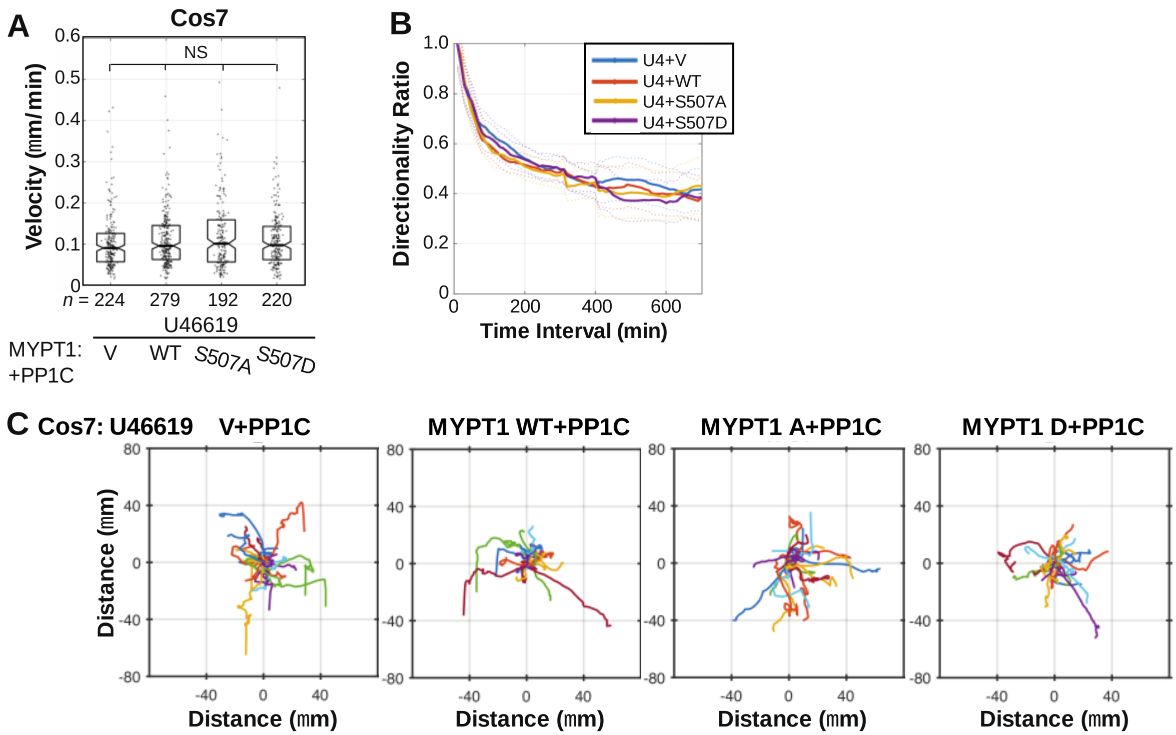


**Figure S5, MYPT1 Ser-507 phosphorylation controls U46689-mediated migration in Cos7 cells. A.** Cell migration velocity distributions of *n* cells treated with Thromboxane agonist U46619, tracked from 3 independent experiments. Boxes span the 25$^{th}$ - 75$^{th}$ distribution. Central horizontal line indicates the median. Notches are 95% C.I. around the median. Distribution *p* values from Kolmogorov-Smirnov test. **B.** Mean directionality of cells analyzed in A., plotted for each time interval. Dashed lines show SEM. **C.** Plots of migrating Cos7 cells. Tracks of the 25 cells with migration speed closest to the median are depicted.

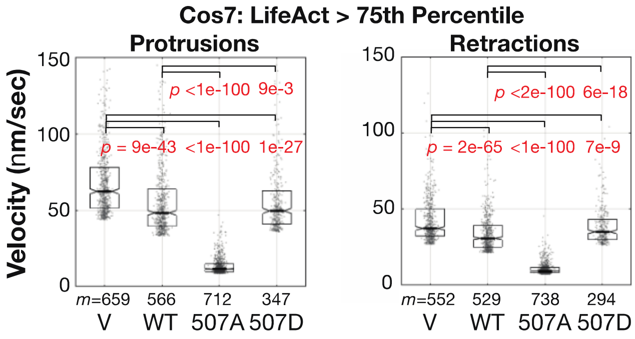


**Figure S6, MYPT1 Ser-507 phosphorylation promotes edge dynamics in Cos7 cells.** Distribution of the top 25% of Cos7 edge events in the corresponding graphs of Figure 8B. *P* values in red for samples with distributions distinct from control DMSO treatment, Kolmogorov-Smirnov test.

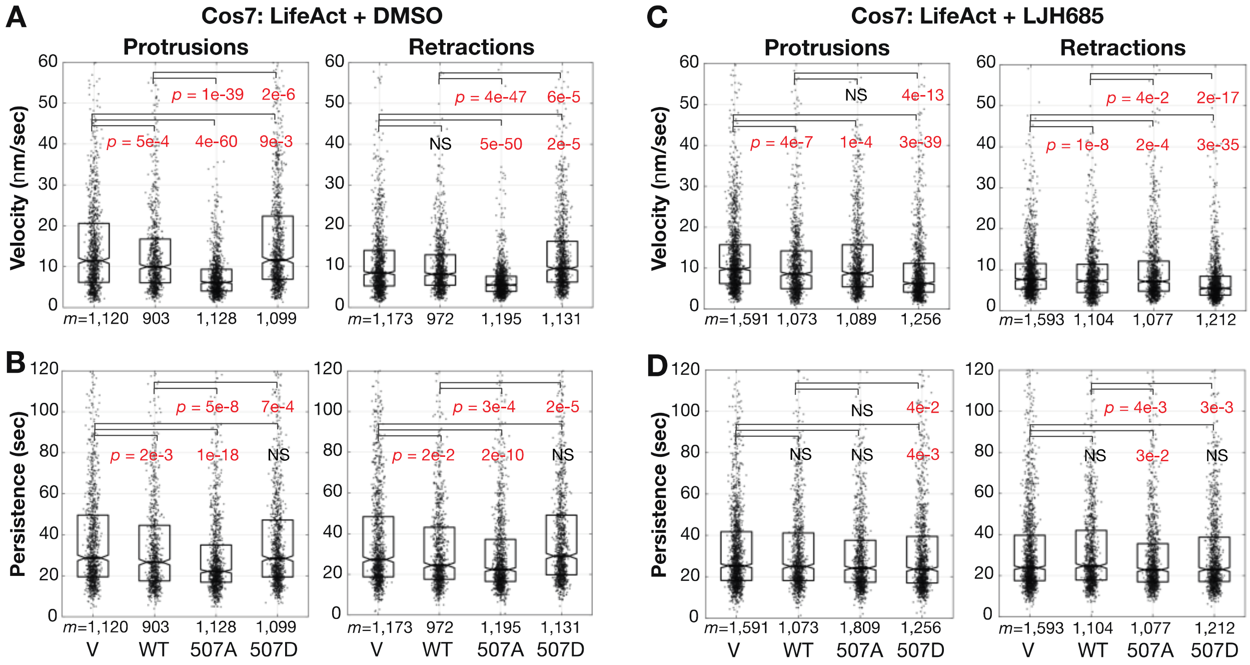


**Figure S7. RSK controls edge dynamics through additional MYPT1-independent mechanisms. A.** Protrusion and retraction velocity distributions of Cos7 cells co-transfected with HA-MYPT1, FLAG-PP1C, and Emerald-Lifeact. **C.** protrusion and retraction persistence distributions of *m* significant protrusion events in *n*=8 cells for Vector, WT (wild-type sequence), and S507A and *n*=4 cell for S507D. Boxes span the 25$^{th}$ - 75$^{th}$ distribution. Each data point is the mean velocity for that event. Central horizontal line indicates the median for all events. Notches are 95% C.I. of median. Samples with distributions distinct from control have *p* values labeled in red, Kolmogorov-Smirnov test. **D.** Model of MYPT1 regulation and action on myosin.
